# Adsorption behavior and prediction model of coalbed methane under the influence of temperature and pressure: a case study of the Zhijin Block, Western Guizhou, South China

**DOI:** 10.1038/s41598-026-52704-3

**Published:** 2026-05-12

**Authors:** Chen Guo, Lingling Jiang, Lingling Lu, Junzhe Gao, Xi Cheng

**Affiliations:** 1 College of Geology and Environment, Xi’an University of Science and Technology, Xi’an, 710054 China; 2 Key Laboratory of Coal Resources Exploration and Comprehensive Utilization，Ministry of Natural Resources, Xi’an, 710021 China; 3 Shaanxi Provincial Key Laboratory of Geological Support for Coal Green Exploitation, Xi’an, 710054 China; 4 State Key Laboratory of Continental Evolution and Early Life, Department of Geology, Northwest University, Xi’an, 710069 China; 5 China Aerial Photogrammetry and Remote Sensing Bureau of China Administration of Coal Geology, Xi’an, 710199 China; 6 Xi’an Coal Aviation Remote Sensing Information Co., LTD, Xi’an, 710100 Shaanxi Province China; 7 Shanxi Geological Exploration Bureau, Geological Team 214 Co., Ltd., Yuncheng, 044000 China

**Keywords:** Deep coalbeds, High rank coals, Isothermal adsorption, Critical depth, Gas adsorption capacity, Prediction model, Energy science and technology, Environmental sciences, Solid Earth sciences

## Abstract

In recent years, significant breakthroughs have been achieved in the exploration and development of deep coalbed methane in northern China. These have revealed marked differences in gas content between deep and shallow coalbeds. The Zhijin Block in western Guizhou, southern China, was one of the earliest regions to achieve commercial CBM development in southern China. As development targets extend progressively to greater depths, the region is now transitioning from shallow beds (< 1000 m) to deep beds (> 1000 m). However, the gas-bearing property and enrichment patterns in these deep coalbeds remain poorly understood. This study focused on the coal-bearing formation of the Longtan Formation in the Zhijin Block to address this issue. Six high-rank coal samples from different regions underwent isothermal adsorption experiments at 30 °C, 45 °C, and 60 °C. The study analyzed the response patterns and key controlling factors of coal sample adsorption behavior to temperature–pressure conditions, establishing a predictive model for adsorption gas content in deep coalbeds. Results indicate: (1) The adsorption behavior of coal samples towards CH₄ conforms to the supercritical Langmuir model. Adsorption capacity decreases with increasing temperature and increases with increasing pressure. (2) A coupled temperature–pressure prediction model for gas adsorption in deep coalbeds was established based on experimental data. Integrating parameters such as the geothermal and fluid pressure gradients revealed that the gas adsorption content initially increases and then decreases with burial depth, indicating the existence of a critical depth in the vertical direction. (3) The spatial distribution characteristics and primary controlling factors of the critical depth for adsorption gas content were elucidated. Both the geothermal gradient and the fluid pressure gradient exhibit negative correlations with the critical depth. (4) Coal adsorption ability exhibits a positive correlation with fixed carbon content, maximum vitrinite reflectance and vitrinite content, and a negative correlation with inertinite content. The research findings provide a theoretical basis for evaluating deep coalbed methane resources, predicting favorable zones, and enabling efficient development in the Zhijin block and other areas of southern China with similar geological conditions.

## Introduction

With the transformation of the global energy structure and the growing demand for clean energy, coalbed methane (CBM) has emerged as a crucial unconventional natural gas resource, its strategic importance increasingly prominent. Currently, China’s exploration and development technologies for shallow CBM resources are maturing. As shallow resources are gradually depleted, the focus of exploration and development will inevitably shift toward deep coalbeds^1,2^. In deep formation environments, the coupled effects of temperature and pressure create a critical depth for coalbed gas adsorption^3^. Above this critical depth, the positive effect of pressure on adsorption dominates, causing adsorption quantity to increase with burial depth. Below the critical depth, the negative effect of temperature on adsorption becomes dominant, causing adsorption quantity to decrease with increasing burial depth. This results in significant changes in the gas content and occurrence state of deep CBM, with its enrichment patterns differing markedly from those in shallow coalbeds^4–6^.

CBM exploration in the Zhijin Block of Guizhou Province commenced in 2009, but relevant research has primarily focused on CBM resources at depths shallower than 1000 m^7^. The Zhijin block features a gently formed coal-bearing syncline structure, with coalbeds that are only slightly deformed and well-preserved coal body structures. It also possesses substantial cumulative coalbed thickness. These characteristics create favorable conditions for the occurrence of CBM resources^8,9^. The Zhijin Block possesses abundant deep CBM resources, but current research on these resources remains limited, hindering the expansion of CBM exploration and development into deeper formation^10^. Compared to shallow CBM, deep CBM exhibits confined hydrogeological conditions and more stable tectonic-geological settings, offering significant potential for high-yield^11,12^. However, prior studies have overlooked the gas-bearing characteristics and key controlling factors of deep coalbeds under high-temperature and high-pressure conditions. Consequently, the enrichment regularity and resource potential of deep CBM in the Zhijin Block remain unclear.

Therefore, coal samples were collected from working faces in six coal mines across four synclines in the Zhijin block. Isothermal adsorption experiments were conducted under varying temperature conditions to establish a predictive model for adsorption gas content under coupled temperature–pressure control. This study systematically analyzed the adsorption behavior of coal samples under changing temperature and pressure conditions and identified the primary controlling factors. The research findings are expected to provide theoretical support for revealing the enrichment patterns of deep CBM and promoting efficient exploration and development of CBM resources in western Guizhou province.

## Study area

**Fig. 1:**
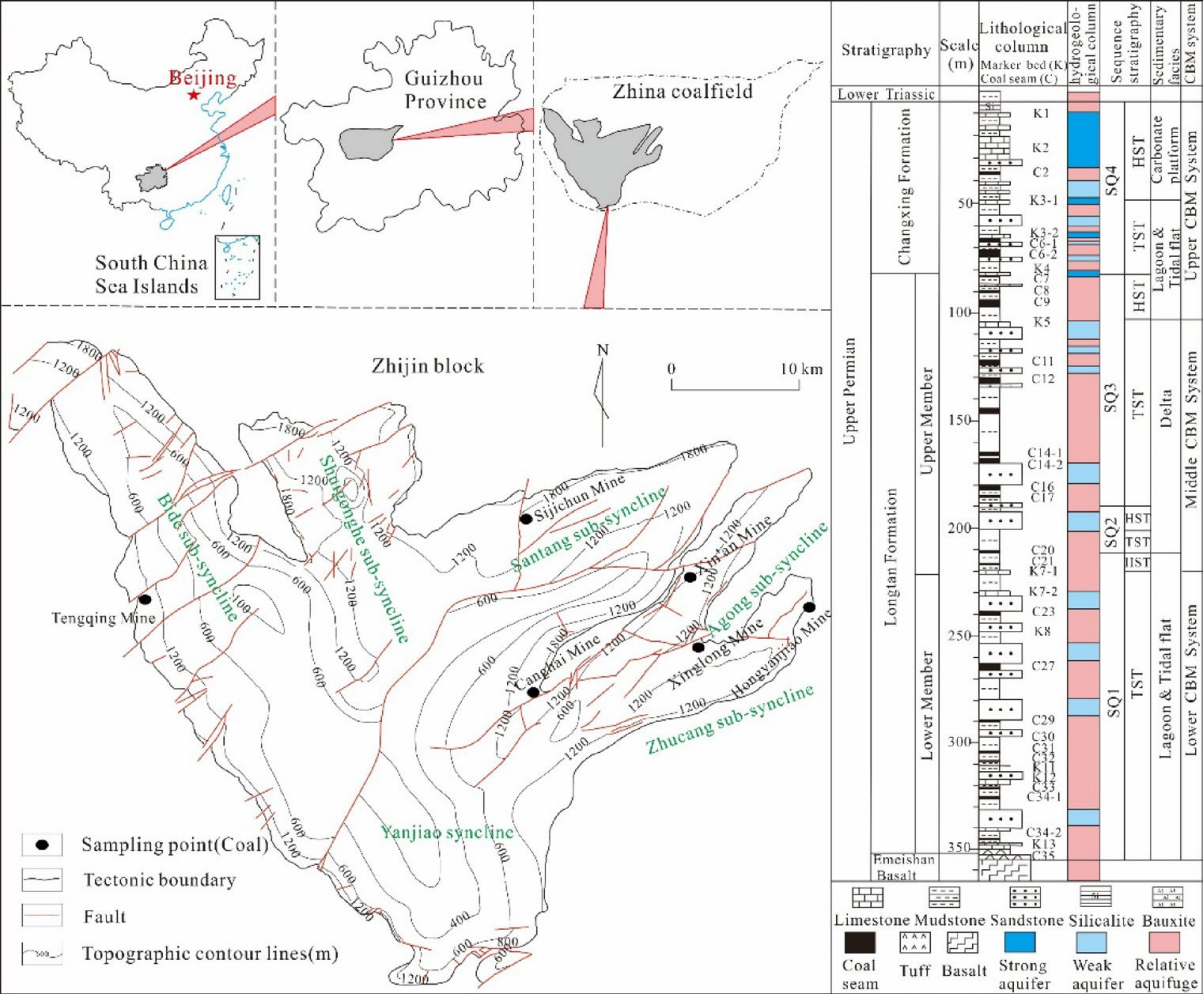
Geological setting of the Zhijin Block.

The Zhijin Block is in the western region of Guizhou Province and represents a significant coal-rich area in southern China (Fig. 1). During its tectonic evolution, this region underwent multiple episodes of compression from various directions, with its tectonic framework stabilizing during the late Yanshanian Orogeny. Three primary structural systems developed: an east-to-west trending system, a northwest-to-southeast trending system, and a northeast-to-southwest trending system. The Zhijin Syncline can be subdivided into five secondary synclines: the Zhucang Sub-Syncline, Agong Sub-Syncline, Santang Sub-Syncline, Bide Sub-Syncline, and Shuigonghe Sub-Syncline^13–15^.

The coal-bearing formation in the study area primarily consists of the Longtan Formation of the Upper Permian, representing a marine-continental transitional facies sequence^16^. The lithology includes mudstone, siltstone, sandstone, limestone, and coalbeds, with a high proportion of fine clastic rocks and dense rock properties^17^. The Longtan Formation ranges in thickness from 196 to 320 m, showing a thinning trend from south to north. It can be divided into two distinct sections. Lower Section: Primarily composed of fine sandstone, mudstone interbedded with limestone and coalbeds. Upper Section: Primarily composed of fine sandstone, siltstone, muddy siltstone, and silty mudstone, rich in plant fossils^18,19^.

The distinctive characteristics of coalbeds can be summarized as “multiple layers, thin individual layers, and substantial cumulative thickness”^20,21^. In terms of coal quality, the coalbeds in this region predominantly belong to the anthracite stage, characterized by high carbon content, low volatile matter, and high vitrinite content. These properties are conducive to the adsorption and storage of CBM^4,22^.

## Samples and methods

Coal samples were collected from the Hongyanjiao Mine and Xinglong Mine in the Zhucang Sub-Syncline, the Canghai Mine and Xin’an Mine in the Agong Sub-Syncline, the Tengqing Mine in the Bide Sub-Syncline, and the Sijichun Mine in the Santang Sub-Syncline within the Zhijin Block. A total of six coal samples were obtained, designated as HYJ, XL, CH, XA, TQ, and SJC respectively. Each sample underwent proximate analysis, maceral analysis, maximum vitrinite reflectance testing, and isothermal adsorption experiments at various temperatures.

### Basic properties testing of samples

Prepare coal samples into 0.1–1 mm size particles for maceral analysis and vitrinite reflectance testing; prepare coal samples into < 0.2 mm size particles for proximate analysis. Proximate analysis was conducted according to the national standard GB/T 30732—2014. Maceral analysis was performed based on ISO 7404-3:2009 and ASTM D2798. Maximum vitrinite reflectance testing was carried out according to the national standard GB/T 6948—2008.

### Isothermal adsorption experimental method

The instrument used in the experiment was an isothermal adsorption analyzer from the College of Geology and Environment at Xi’an University of Science and Technology. The instrument’s temperature control accuracy is ± 0.1 °C, and its pressure control accuracy is ± 0.1 MPa.

Operational Procedure:

1. Coal Sample Preparation: Following the standard High-Pressure Isothermal Adsorption Test Method for Coal (GB/T 19560–2025), prepare the coal sample into particle sizes ranging from 0.25 mm to 0.18 mm. Perform equilibrium water treatment on coal samples according to the standard (MT/T 1157–2011).
2. Pre-experiment Preparation: ① Equipment leak detection: Fill the sample cell, reference cell, and connecting piping with helium. Maintain a pressure exceeding 3 MPa for 12 h to ensure no pressure change occurs. ② Sample loading: Accurately weigh the balanced water coal sample and load it into the sample cell, ensuring uniform filling and avoiding voids. ③ Degassing: After the experimental temperature stabilizes, activate the vacuum pump to initiate evacuation. Subsequently, shut off the vacuum pump, inlet valve, and balancing valve. ④ Free Space Volume Calibration: Open the helium cylinder and inlet valve to fill the reference cell with helium. Adjust the pressure to 2 MPa, close the inlet valve, and record the data after stabilization. Open the communication valve and wait for system pressure equilibrium (stable pressure gauge reading). Record the equilibrium pressure at this point. Repeat the gas filling and data collection four times. Calculate the average free space volume in the sample cell.
3. Isothermal adsorption experiment: Methane is introduced into the sample cell, with pressure incrementally increased. At each pressure point, the sample is held for 12 h until adsorption equilibrium is reached. The equilibrium pressure and volume are recorded.
4. Repeat the experiment: Replace the coal sample and repeat the above steps to sequentially complete methane isothermal adsorption experiments for all six coal samples under different temperature and pressure conditions.
5. According to the ideal gas equation of state, the adsorption volume V_ads_ can be expressed as:
  1 $${\mathrm{V}}_{\mathrm{a}\mathrm{d}\mathrm{s}}=\frac{\left({\mathrm{P}}_{1}-{\mathrm{P}}_{2}\right)\times {\mathrm{V}}_{\mathrm{f}\mathrm{r}\mathrm{e}\mathrm{e}}}{\mathrm{Z}\mathrm{R}\mathrm{T}}\times \frac{1}{\mathrm{m}}\times 22400$$

where, Z represents the gas compression factor, dimensionless; R denotes the universal gas constant, equal to 8.314 J/(mol·K); T is the absolute temperature, K; m is the mass of the coal sample, g; P_1_ is the pre-equilibrium pressure, MPa; P_2_ is the post-equilibrium pressure, MPa; V_free_ is the free phase volume, cm^3^; V_ads_ is the adsorption quantity, cm^3^/g. Calculate the measured excess adsorption using the above equation; 22400 is the molar volume of a gas under standard conditions (0 °C, 1 atm), in cm^3^/mol.

### Supercritical gas isothermal adsorption model

This study investigates six coal samples based on the extended Langmuir supercritical methane excess adsorption model. According to the Langmuir equation, the absolute adsorption quantity V_ab_ can be expressed as^23^:

2 $${\mathrm{V}}_{\mathrm{a}\mathrm{b}}=\frac{{\mathrm{PV}}_{\mathrm{L}}}{\mathrm{P}+{\mathrm{P}}_{\mathrm{L}}}$$

where, V_L_ denotes the Langmuir volume, cm^3^/g; P represents the equilibrium pressure, MPa; P_L_ denotes the Langmuir pressure, MPa.

The experimentally measured adsorption quantity is the excess adsorption quantity V_ex_. By introducing the excess adsorption quantity correction factor (1-ρ_g_/ρ_a_), the extended Langmuir supercritical methane excess adsorption model (S-L model) is derived, expressed as^24^:

3 $${\mathrm{V}}_{\mathrm{e}\mathrm{x}}=\frac{{\mathrm{PV}}_{\mathrm{L}}}{\mathrm{P}+{\mathrm{P}}_{\mathrm{L}}}\left(1-\frac{{\uprho}_{\mathrm{g}}}{{\uprho}_{\mathrm{a}}}\right)$$

where, ρ_g_ represents the density of methane in the free gas phase, g/cm^3^; ρ_a_ denotes the density of methane in the adsorbed phase, g/cm^3^. The calculation formula for ρ_g_ is:

4 $${\uprho}_{\mathrm{g}}=\frac{\mathrm{P}\mathrm{M}}{\mathrm{Z}\mathrm{R}\mathrm{T}}$$

where, P represents the coal reservoir pressure, MPa; M denotes the molar mass of the gas, g/mol; Z is the gas compression factor, dimensionless; R is the universal gas constant, equal to 8.314 J/(mol·K); T is the thermodynamic temperature, K.

**Fig. 2:**
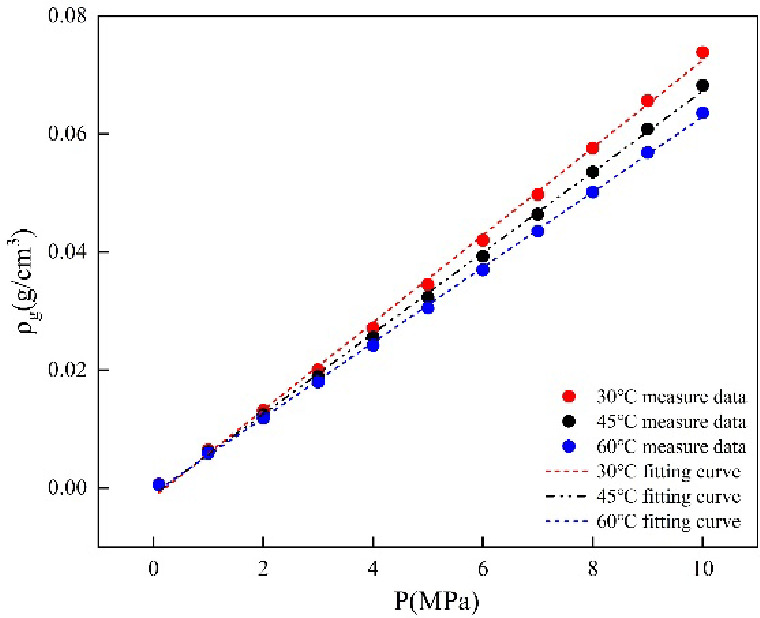
The relationship between the density of methane in the free gas phase and changes in temperature and pressure.

As shown in Fig. 2, the density of free-phase methane increases significantly with rising pressure and decreases with rising temperature. These characteristic underscores the necessity of performing adsorption correction.

Determining the adsorbed-phase density is crucial for model fitting and adsorption quantity calibration. According to research by Ozawa et al.^25^, the adsorbed-phase density and boiling point density follow a specific empirical formula:

5 $${\uprho}_{\mathrm{a}}={\uprho}_{\mathrm{b}}\times \mathrm{e}\mathrm{x}\mathrm{p}\left[-0.0025\times \left(\mathrm{T}-{\mathrm{T}}_{\mathrm{b}}\right)\right]$$

where, ρ_a_ represents the methane adsorbed-phase density, ρ_b_ denotes the density of methane at its boiling point, with a value of 0.424 g/cm^3^ ^26^; T_b_ denotes the boiling point temperature of methane, with a value of − 161.5 °C. Calculations yielded the following methane adsorbed-phase densities: 0.263 g/cm^3^ at 30 °C, 0.253 g/cm^3^ at 45 °C, and 0.244 g/cm^3^ at 60 °C.

## Results

### Basic properties of coal samples

The moisture content of the six coal samples ranged from 0.47% to 2.15%, with sample HYJ exhibiting a relatively high moisture content of 2.15%, while the other samples had lower moisture levels. Volatile matter content varied between 6.18% and 18.19%, with sample TQ showing a relatively high volatile matter yield of 18.19%, while the volatile matter content of the other samples was less than 10%. The ash content ranged from 11.57% to 25.87%. The fixed carbon content ranged from 63.41% to 82.35% (Table 1). The coal samples exhibit low volatile matter yield and high carbon content, indicating that the coalbeds possess favorable adsorption properties capable of storing substantial amounts of CBM^27,28^.

**Table 1:** Macerals and proximate analysis results of coal samples.

Coal samples number	Moisture/%	Volatile matter/%	Ash content/%	Fixed carbon/%	Organic components/%		Inorganic mineral content	Maximum vitrinite reflectance/%
					vitrinite	inertinite		
HYJ	2.15	7.27	25.87	67.23	82.20	2.89	9.99	2.89
XL	0.78	6.18	11.57	82.35	83.01	2.98	11.16	2.98
CH	0.78	7.64	24.47	68.03	66.80	2.59	12.21	2.59
XA	1.48	7.22	13.71	79.3	75.60	2.73	8.15	2.73
TQ	0.47	18.19	18.59	63.41	57.45	1.75	8.09	1.75
SJC	0.62	6.78	17.19	76.13	80.60	2.64	10.27	2.64

Maceral and maximum vitrinite reflectance tests on six coal samples indicate (Table 1): vitrinite content ranged from 57.45% to 83.01%, inertinite content ranged from 5.83% to 34.46%. The inorganic mineral content ranges from 8.09% to 12.21%. The maximum vitrinite reflectance varies from 1.75% to 2.98%. The study area is dominated by anthracite, with low-volatile bituminous coal forming only in the Bide Sub-syncline^29^.

### Isothermal adsorption properties

The experiment was conducted at three temperature points: 30°C, 45°C, and 60°C. At each temperature point, isothermal adsorption experiments were performed under varying pressures within the range of 0.1 to 10.0 MPa.

The S-L model was applied to fit the measured methane isothermal adsorption data. The fitting results are presented in Table 2, with determination coefficients R^2^ all exceeding 0.930. This indicates that the S-L model is suitable for describing the adsorption process of methane by coal samples.

**Table 2:** Fitting results of methane isothermal adsorption experiments.

Coal samples	30℃			45℃			60℃		
	V_L_(cm^3^/g)	P_L_(MPa)	R^2^	V_L_(cm^3^/g)	P_L_(MPa)	R^2^	V_L_(cm^3^/g)	P_L_(MPa)	R^2^
HYJ	30.908	2.238	0.957	29.618	2.909	0.980	24.564	1.998	0.931
XL	35.620	3.446	0.984	33.536	4.154	0.982	26.353	2.969	0.999
CH	29.299	2.207	0.955	27.246	2.346	0.966	24.646	2.103	0.984
XA	36.084	3.145	0.955	29.225	3.028	0.957	23.928	2.119	0.954
TQ	25.153	2.253	0.967	20.830	2.179	0.970	18.890	2.410	0.970
SJC	35.050	3.279	0.971	30.900	3.046	0.971	26.532	2.421	0.986

**Fig. 3:**
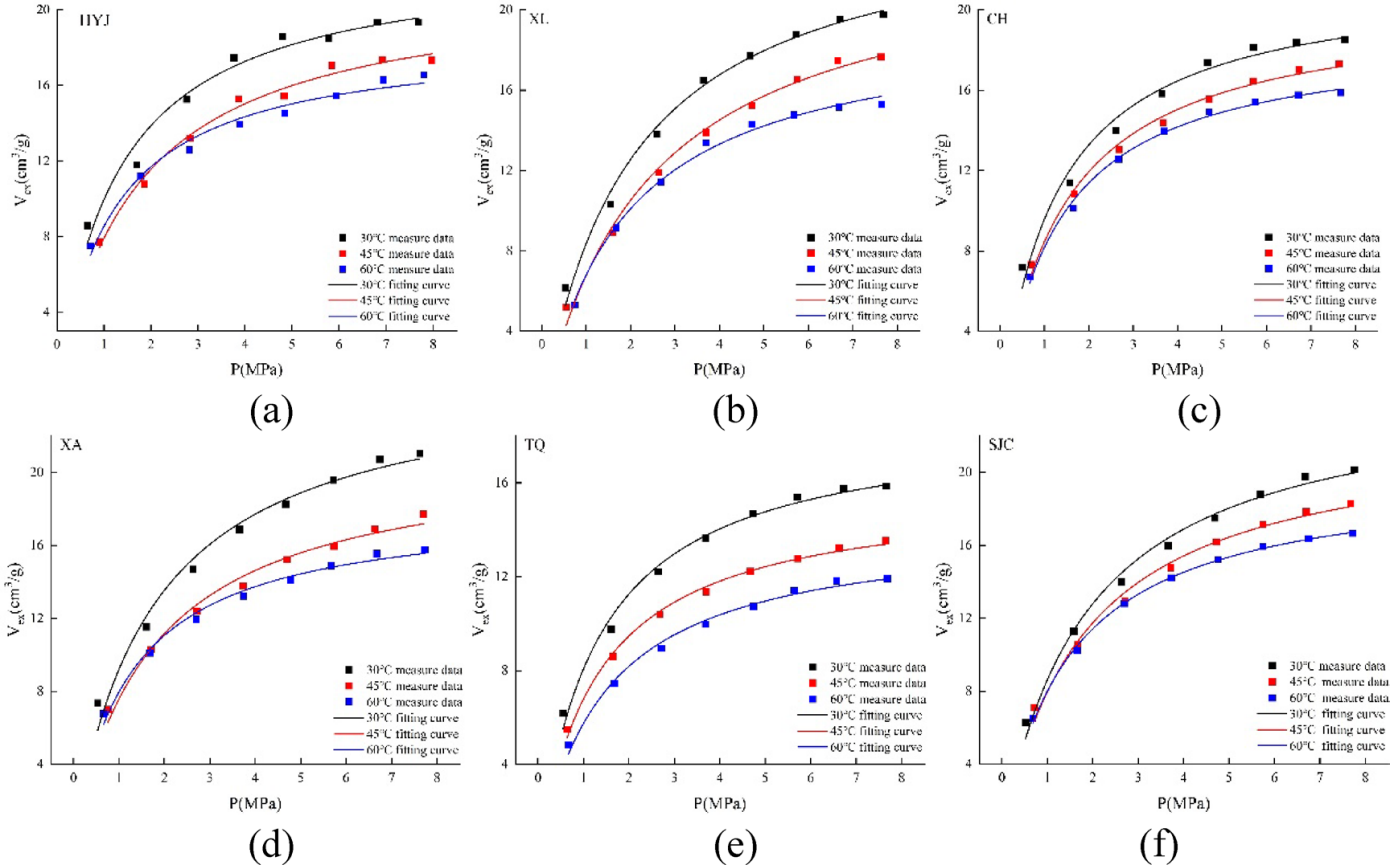
Fitted curve of measured adsorption quantity Vex for coal samples. (**a**) HYJ, (**b**) XL, (**c**) CH, (**d**) XA, (**e**) TQ, (**f**) SJC.

The fitting results indicate (Fig. 3): (1) For all six coal samples, adsorption capacity increased rapidly during the low-pressure stage (0–6 MPa). During the high-pressure stage (6–10 MPa), the adsorption quantity continued to increase gradually, but the increase rate decreased progressively, with adsorption gradually approaching saturation; (2) As temperature increased, the CH₄ adsorption capacity of all six samples gradually decreased.

**Fig. 4:**
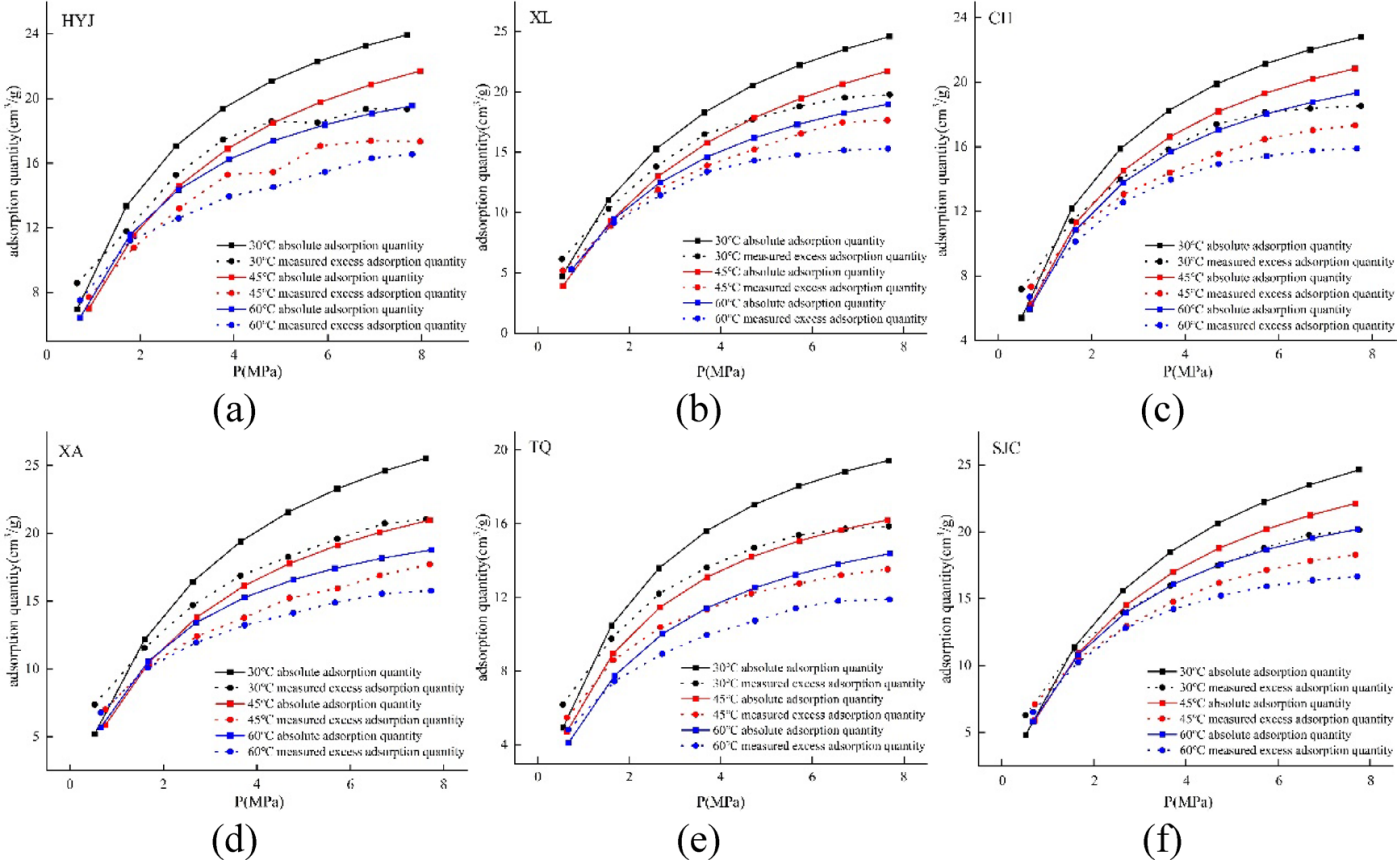
Measured excess adsorption vs absolute adsorption curve for coal samples. (**a**) HYJ, (**b**) XL, (**c**) CH, (**d**) XA, (**e**) TQ, (**f**) SJC.

The absolute adsorption quantity was calculated using Eq. (2). By comparing the measured excess adsorption quantity with the absolute adsorption quantity for the coal samples under different temperature and pressure conditions (Fig. 4), the following information can be obtained:

(1) At low pressures, the measured and absolute adsorption data overlap, with both increasing rapidly with pressure. At medium-to-high pressures, the rate of increase for measured excess adsorption quantity slows significantly, deviating from and falling below absolute adsorption quantity. This further demonstrates the necessity of introducing an excess adsorption correction model. (2) As adsorption pressure increases, the gas content of the coal samples for both measured excess adsorption quantity and absolute adsorption quantity continuously rises, though their rates of increase gradually decrease. (3) Throughout the experimental pressure range, adsorption capacity decreased continuously with increasing temperature. For example, taking the HYJ coal sample as an example: at an experimental temperature of 30 °C, absolute adsorption quantity increased by 12.41 cm^3^/g when adsorption pressure rose from 0 to 3 MPa; however, when pressure increased from 4 to 7 MPa, absolute adsorption quantity increased by only 2.86 cm^3^/g. At a pressure of 4 MPa, increasing the temperature from 30 °C to 45 °C resulted in a 2.593 cm^3^/g decrease in the absolute adsorption quantity of this coal sample. Increasing the temperature from 45°C to 60°C, led to a further reduction of 1.1 cm^3^/g in absolute adsorption quantity.

## Discussion

### Prediction model for adsorption gas content

#### Prediction model based on temperature and pressure conditions

Using the S-L model to fit the measured adsorption quantity yielded V_L_ and P_L_. Based on Eq. (5), the adsorbed-phase density ρ_a_ was calculated, revealing that both parameters are functions of temperature (Table 3). Therefore, temperature T was further introduced into Eq. (2), resulting in:

**Table 3:** Relationship equations between isothermal adsorption constants and temperature for six coal samples.

Coal samples	Relationship equations	Determination coefficients
HYJ	V_L_ = 95.63824–0.21146 T	0.894
	P_L_ = -11.31139 + 0.0447 T	0.990
XL	V_L_ = 130.10971–0.30889 T	0.908
	P_L_ = -10.8605 + 0.04719 T	0.999
CH	V_L_ = 76.40527–0.15509 T	0.995
	P_L_ = -0.59956 + 0.00926 T	0.990
XA	V_L_ = 158.65329–0.40518 T	0.995
	P_L_ = 22.28895–0.06054 T	0.990
TQ	V_L_ = 88.02908–0.20872 T	0.954
	P_L_ = -2.71957 + 0.0154 T	0.990
SJC	V_L_ = 121.16404–0.28394 T	0.999
	P_L_ = 12.02063–0.02862 T	0.935

6 $${\mathrm{V}}_{\mathrm{a}\mathrm{b}}=\frac{{\mathrm{V}}_{\mathrm{L}}\left(\mathrm{T}\right)\mathrm{P}}{\mathrm{P}+{\mathrm{P}}_{\mathrm{L}}\left(\mathrm{T}\right)}$$

Substituting the relationship between V_L_, P_L_ and temperature into Eq. (6) produces a theoretical model of the adsorption gas content in deep coal reservoirs (Table 4). This model can be used to predict adsorption gas content in deep coal reservoirs and analyze how it varies with temperature and pressure.

**Table 4:** Prediction models for absolute adsorption quantity of coal samples.

Coal samples	Prediction models for absolute adsorption
HYJ	V_ab_ = $\frac{(95.63824-0.21146\mathrm{T})\mathrm{P}}{\mathrm{P}+(-11.31139+0.0447\mathrm{T})}$
XL	V_ab_ = $\frac{(130.10971-0.30889\mathrm{T})\mathrm{P}}{\mathrm{P}+(-10.8605+0.04719\mathrm{T})}$
CH	V_ab_ = $\frac{(76.40527-0.15509\mathrm{T})\mathrm{P}}{\mathrm{P}+(-0.59956+0.00926\mathrm{T})}$
XA	V_ab_ = $\frac{(158.65329-0.40518\mathrm{T})\mathrm{P}}{\mathrm{P}+(22.28895-0.06054\mathrm{T})}$
TQ	V_ab_ = $\frac{(88.02908-0.20872\mathrm{T})\mathrm{P}}{\mathrm{P}+(-2.71957+0.0154\mathrm{T})}$
SJC	V_ab_ = $\frac{(121.16404-0.28394\mathrm{T})\mathrm{P}}{\mathrm{P}+(12.02063-0.02862\mathrm{T})}$

V_ab_ denotes the absolute adsorption quantity, cm^3^/g; T represents the temperature of the coal reservoir, K; P indicates the reservoir pressure, MPa.

#### Depth-based prediction model

To reveal the vertical variation pattern of adsorption gas content, the prediction model (Table 4) established is coupled with coalbed depth. The functional relationship between temperature, pressure, and coalbed depth is as follows:

7 $$\mathrm{P}={\mathrm{P}}_{0}+{\mathrm{P}}_{\mathrm{H}}\times \mathrm{D}$$

8 $$\mathrm{T}={\mathrm{T}}_{0}+{\mathrm{T}}_{\mathrm{H}}\times \mathrm{D}$$

where P_0_ represents surface pressure, typically set at 0.1 MPa; T_0_ denotes surface temperature, set at 303.15 K; D indicates depth, meters. P_H_ is the coal reservoir fluid pressure gradient, MPa/m, while T_H_ is the coal reservoir geothermal gradient, °C/m. The P_H_ and T_H_ values for the six coal sample locations are shown in Table 5^30^:

**Table 5:** Geothermal gradient and fluid pressure gradient at sample locations.

Coal samples	Geothermal gradient T_H_(°C/m)	Fluid pressure gradient P_H_(MPa/m)
HYJ	0.024	0.008
XL	0.027	0.011
CH	0.018	0.013
XA	0.024	0.012
TQ	0.022	0.006
SJC	0.016	0.007

Substituting Eqs. (7) and (8) into the adsorption gas content prediction model yields the relationship between methane adsorption quantity and burial depth for six coal samples (Table 6).

**Table 6:** Relationship equations between methane adsorption quantity and burial depth for six coal samples.

Coal samples	Relationship equations between methane adsorption and burial depth
HYJ	V_ab_ = $\frac{[95.63824-0.21146(303.15+0.024\times \mathrm{D})](0.1+0.008\times \mathrm{D})}{(0.1+0.008\times \mathrm{D})+[-11.31139+0.0447(303.15+0.024\times \mathrm{D})]}$
XL	V_ab_ = $\frac{[130.10971-0.30889(303.15+0.027\times \mathrm{D})](0.1+0.011\times \mathrm{D})}{(0.1+0.011\times \mathrm{D})+[-10.8605+0.04719(303.15+0.027\times \mathrm{D})]}$
CH	V_ab_ = $\frac{[76.40527-0.15509(303.15+0.018\times \mathrm{D})](0.1+0.013\times \mathrm{D})}{(0.1+0.013\times \mathrm{D})+[-0.59956+0.00926(303.15+0.018\times \mathrm{D})]}$
XA	V_ab_ = $\frac{[158.65329-0.40518(303.15+0.024\times \mathrm{D})](0.1+0.012\times \mathrm{D})}{(0.1+0.012\times \mathrm{D})+[22.28895-0.06054(303.15+0.024\times \mathrm{D})]}$
TQ	V_ab_ = $\frac{[88.02908-0.20872(303.15+0.022\times \mathrm{D})](0.1+0.006\times \mathrm{D})}{(0.1+0.006\times \mathrm{D})+[-2.71957+0.0154(303.15+0.022\times \mathrm{D})]}$
SJC	V_ab_ = $\frac{[121.16404-0.28394(303.15+0.016\times \mathrm{D})](0.1+0.007\times \mathrm{D})}{(0.1+0.007\times \mathrm{D})+[12.02063-0.02862(303.15+0.016\times \mathrm{D})]}$

**Fig. 5:**
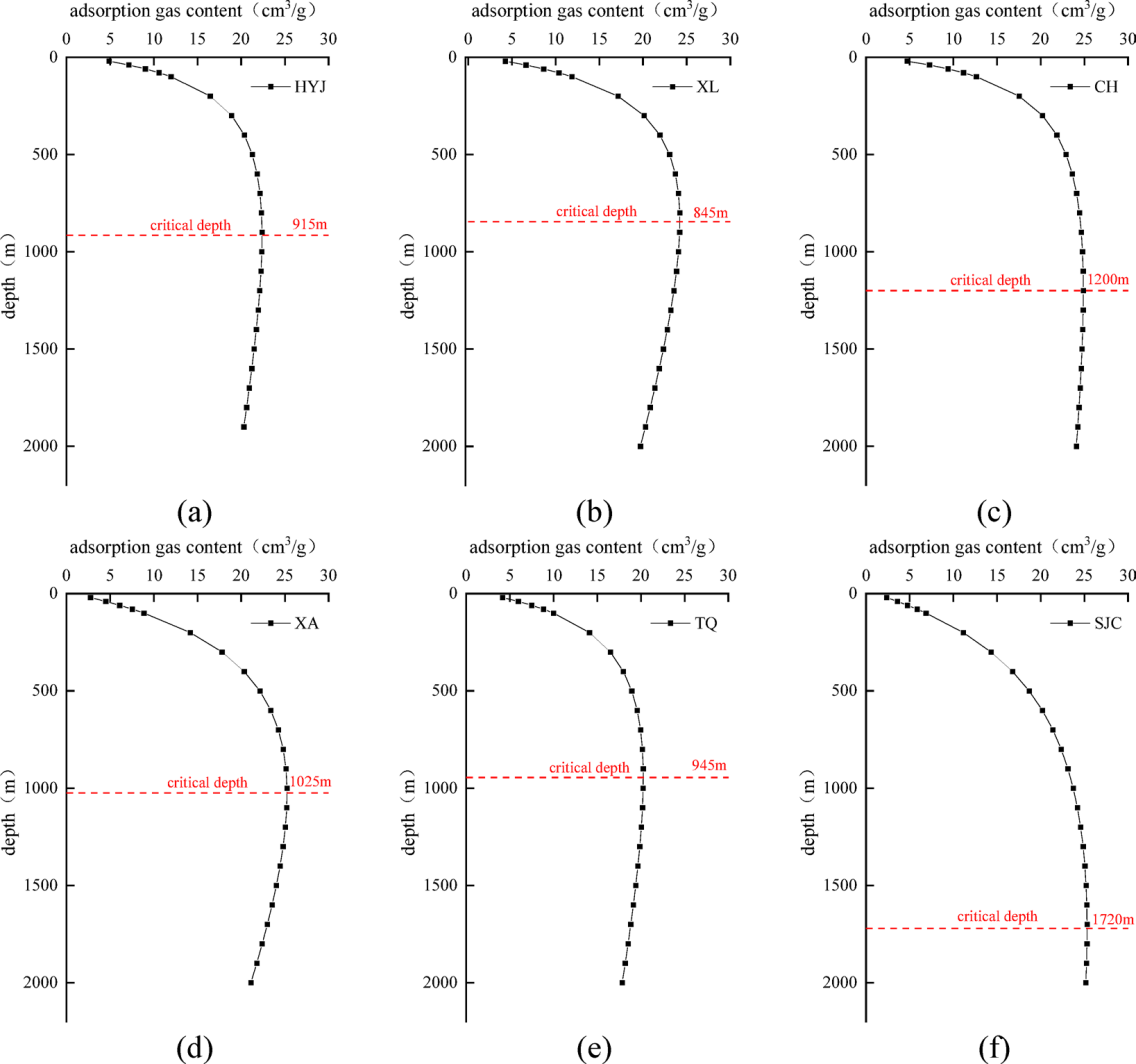
Variation of adsorption gas content and critical depth for each coal sample with burial depth. (**a**) HYJ, (**b**) XL, (**c**) CH, (**d**) XA, (**e**) TQ, (**f**) SJC.

As shown in Fig. 5, the adsorption gas content exhibits a non-monotonic trend with depth. Initially, it increases rapidly, then slows down, peaks and gradually decreases. Therefore, a critical depth exists for the six coal samples, with critical depths of 915 m, 845 m, 1200 m, 1025 m, 945 m and 1720 m, respectively. Above the critical depth, the adsorption gas content increases with depth due to the pressure-enhanced adsorption effect. Below the critical depth, however, the adsorption gas content gradually declines, indicating that, as the formation temperature rises, its inhibitory effect on adsorption begins to outweigh the pressure-enhanced adsorption effect. The critical depth represents the upper limit for effective accumulation of free gas in coal measures. Beyond this depth, coal measure reservoirs possess the potential for free gas accumulation. Therefore, critical depth serves as the basis for predicting the resource potential of deep CBM, and can also provide a fundamental basis for evaluating the co-existence potential of coal measure gases, as well as for designing technical schemes for co-exploration and co-production.

**Fig. 6:**
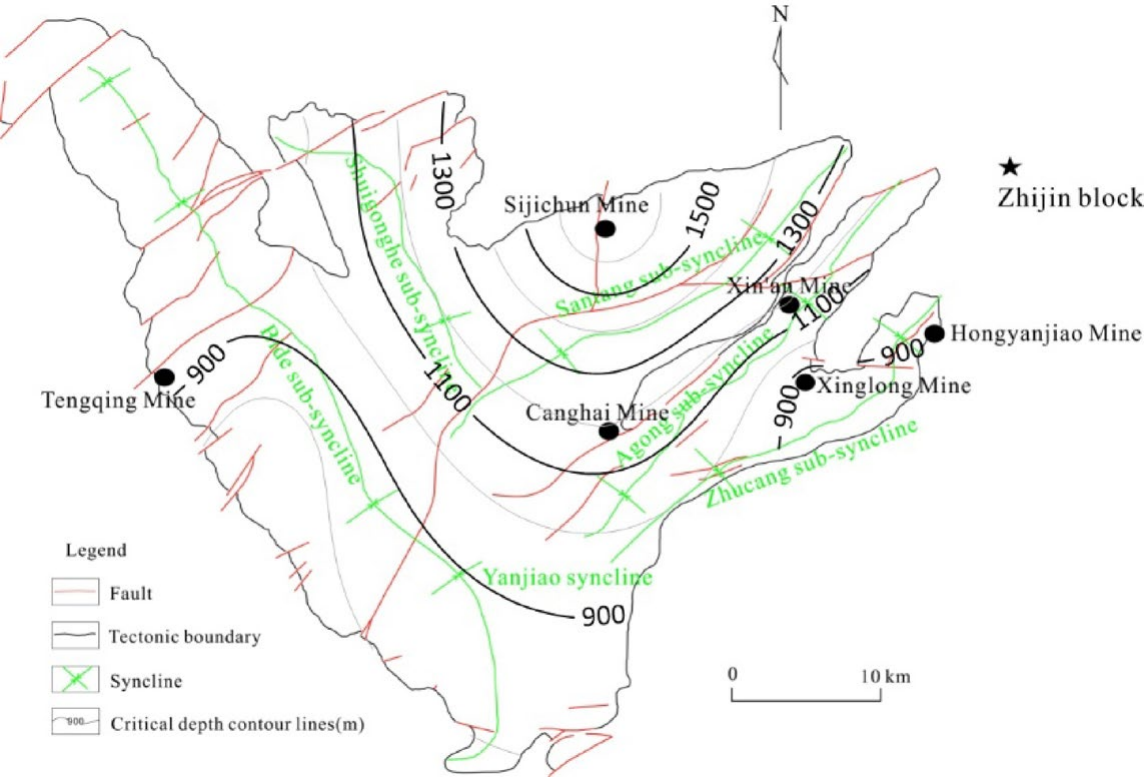
Critical depth contour map.

A critical depth contour map of the Zhijin Block was constructed based on the critical depth values from six coal samples. The critical depth in the study area ranges from 800 to 1750 m, exhibiting significant variation spatially. Overall, there is a gradual increase from southwest to northeast (Fig. 6). Of these areas, the Santang Sub-Syncline has the greatest critical depth for adsorption gas content. The application value of Fig. 6 is reflected in the following aspects: (1) The critical depths for the Bide and Zhucang synclines are shallow, allowing free gas to accumulate at around 900 m. These two synclines are favorable areas for co-exploration and co-production of coal measure gas. (2) The critical depths for the Shuigonghe, Santang, and Agong synclines are deep, requiring free gas accumulation to exceed at least 1,100 m, and even surpass 1,300 m in the Santang syncline. (3) The critical depth only indicates the temperature and pressure conditions under which adsorbed gas transforms into free gas. Whether free gas can effectively accumulate also depends on favorable preservation conditions. Future research should focus on the preservation conditions for coal measure gas enrichment.

### Factors affecting adsorption

#### Coal rank

The adsorption properties of coal are significantly influenced by its metamorphic grade, while maximum vitrinite reflectance, content of fixed carbon and volatile matter yield are three key indicators that characterize coal rank^31^.

**Fig. 7:**
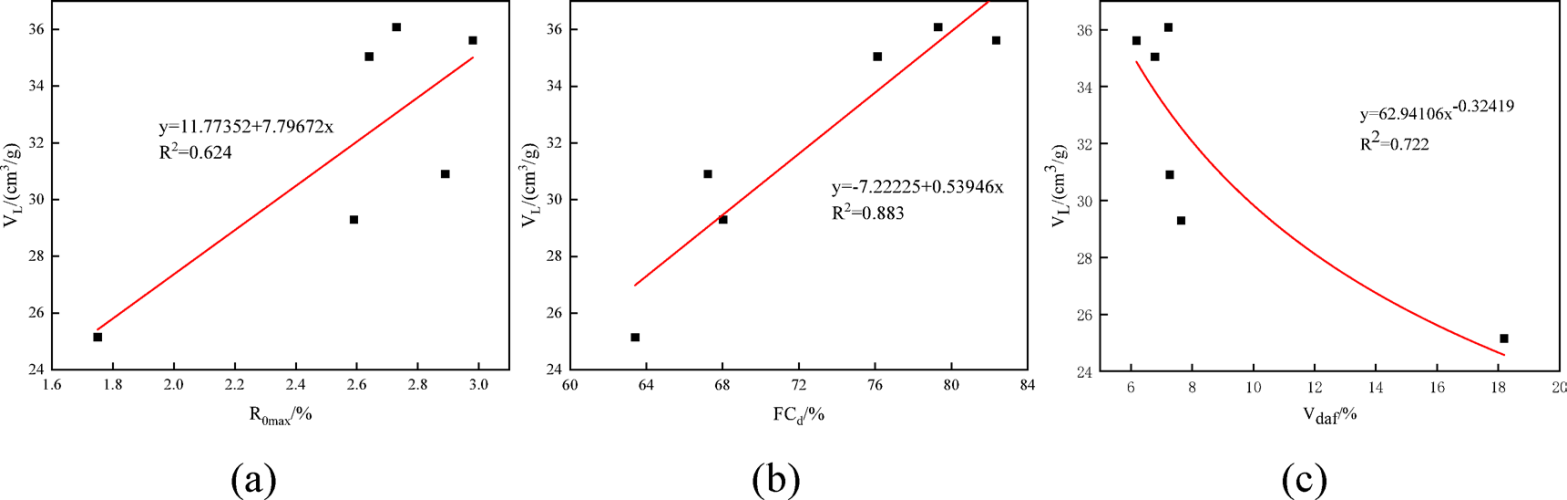
(**a**) Relationship between maximum vitrinite reflectance and Langmuir volume; (**b**) Relationship between content of fixed carbon and Langmuir volume; (**c**) Relationship between volatile matter yield and Langmuir volume.

There is a gradually increasing trend in V_L_ with rising maximum vitrinite reflectance. However, the study area is dominated by high-rank coal, which has limited variation in the degree of coalification. This results in a weakened correlation between V_L_ and vitrinite reflectance^32,33^, with determination coefficient R^2^ of 0.624. The metamorphic grade of the coal samples supports the strong adsorption capacity (Fig. 7a).

The fixed carbon content of coal samples exhibits a positive correlation with Langmuir volume^34^ (Fig. 7b), with determination coefficient R^2^ of 0.883, indicating a significant correlation.

The volatile matter yield exhibits a Power function relationship with Langmuir volume (Fig. 7c), with determination coefficient R^2^ of 0.722. Overall, coal rank is the fundamental controlling factor for the adsorption of coal samples in the study area.

#### Macerals

**Fig. 8:**
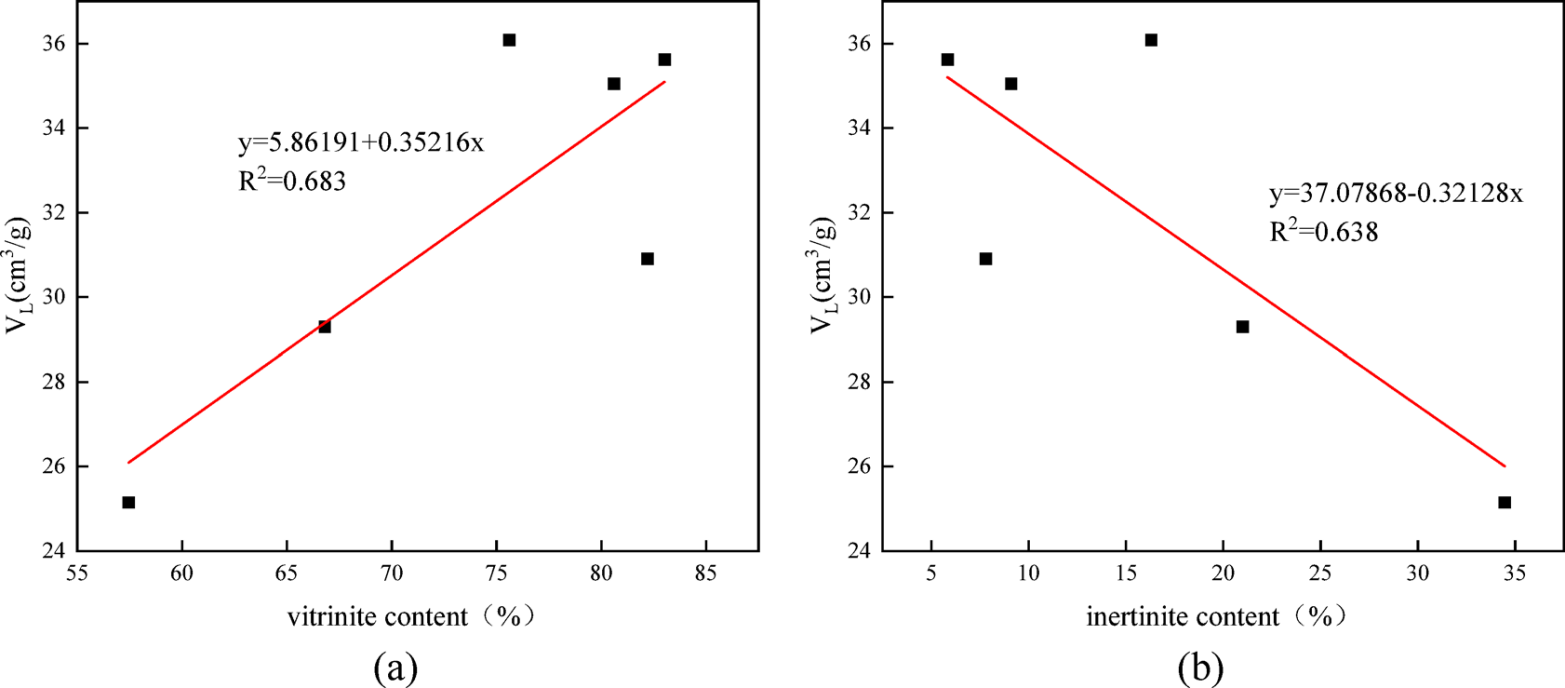
(**a**) Relationship between vitrinite content and Langmuir volume; (**b**) Relationship between inertinite content and Langmuir volume.

For similar coal ranks, maceral composition is the key intrinsic factor. During evolution, vitrinite forms abundant micropores, whose enormous specific surface area provides a large number of adsorption sites and thereby enhances adsorption^35^. The vitrinite content of the six coal samples in this study showed a significant positive correlation with the Langmuir volume, with determination coefficient R^2^ of 0.683(Fig. 8a). Inertinite content exhibits a negative correlation with Langmuir volume, with a determination coefficient R^2^ of 0.638. This suggests that inertinite reduces coal adsorption, probably due to its well-developed macropores (Fig. 8b).

#### Temperature

**Fig. 9:**
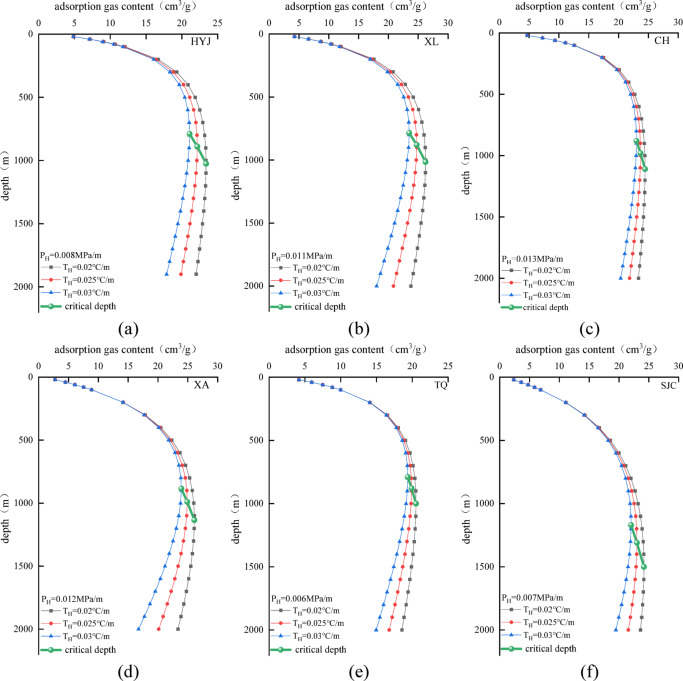
Relationship between adsorption gas content and burial depth under geothermal gradient constraints. (**a**) HYJ, (**b**) XL, (**c**) CH, (**d**) XA, (**e**) TQ, (**f**) SJC.

With a fixed fluid pressure gradient, three different geothermal gradients (0.020 °C/m, 0.025 °C/m and 0.030 °C/m) were applied to plot the relationship between the adsorption gas content and the depth of the coalbed under various geothermal conditions. The results show that, at the same burial depth, the adsorption gas content in coalbeds decreases as the geothermal gradient increases. Above the critical depth, the geothermal gradient has a negligible effect on coalbed adsorption gas content. However, below the critical depth, the difference in adsorption gas content across different temperature gradients gradually increases, indicating that the influence of temperature on adsorption gas content progressively strengthens (Fig. 9).

**Fig. 10:**
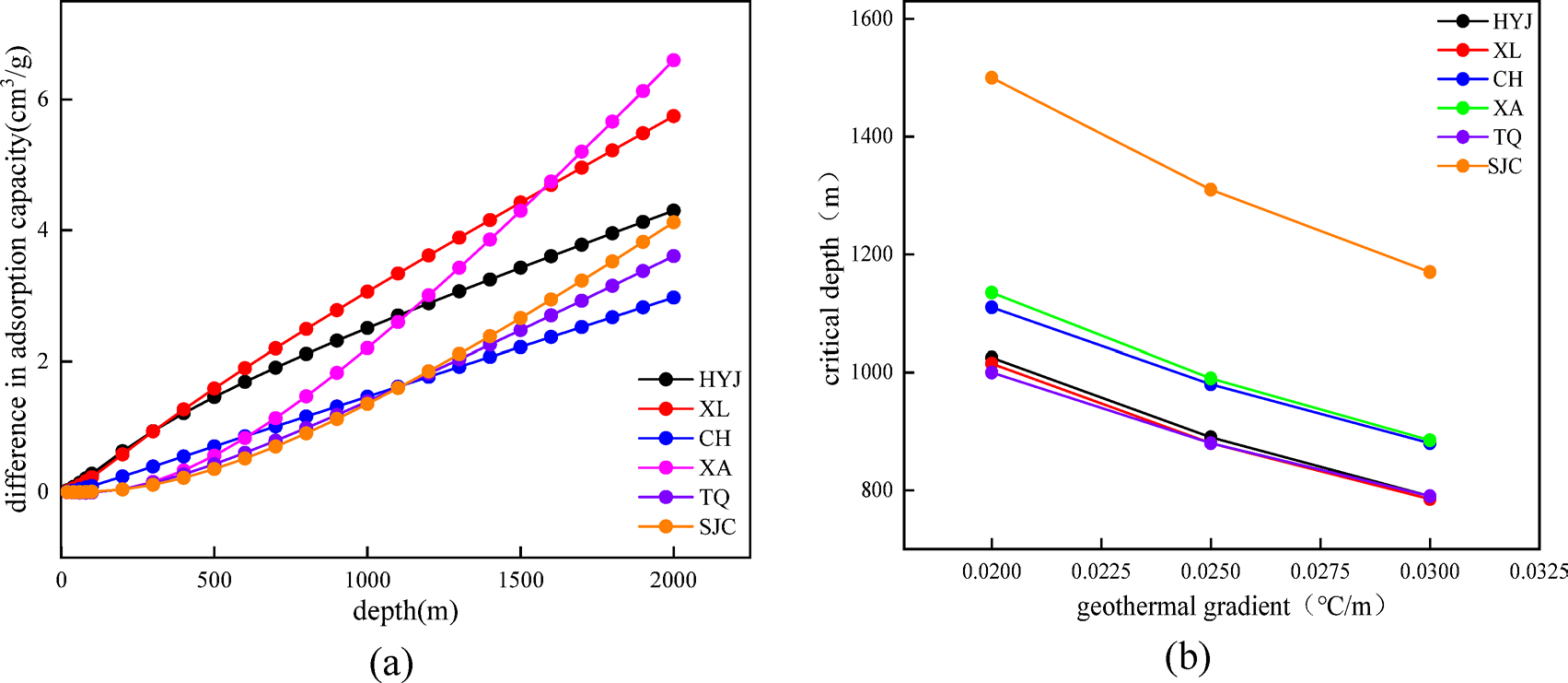
(**a**) Variation in the difference in absolute adsorption capacity with depth under different ground geothermal gradients (0.020 °C/m, 0.030 °C/m), (**b**) Relationship between geothermal gradient and critical depth.

For the six coal samples, the difference in absolute adsorption quantity under two geothermal gradient conditions (0.02 °C/m and 0.03 °C/m) showed a monotonically increasing trend with burial depth (Fig. 10a); at a burial depth of 2,000 m, this difference reached 2.8–6.5 cm^3^/g. This indicates that the influence of geothermal gradient differences on the content of adsorbed gas gradually increases with burial depth, and should not be overlooked in the evaluation of deep coal seams.

Further investigation reveals the impact of the geothermal gradient on the critical depth (Fig. 10b). As the geothermal gradient increases from 0.02 °C/m to 0.03 °C/m, the critical depths of all six coal samples shift to shallower depths. For example: The critical depth of the HYJ coal sample decreases from 1025 to 790 m as the geothermal gradient increases. Therefore, the geothermal gradient is a significant factor influencing the occurrence of CBM in the study area. increasing geothermal gradient leads to shallower critical depths in coalbeds.

#### Pressure

**Fig. 11:**
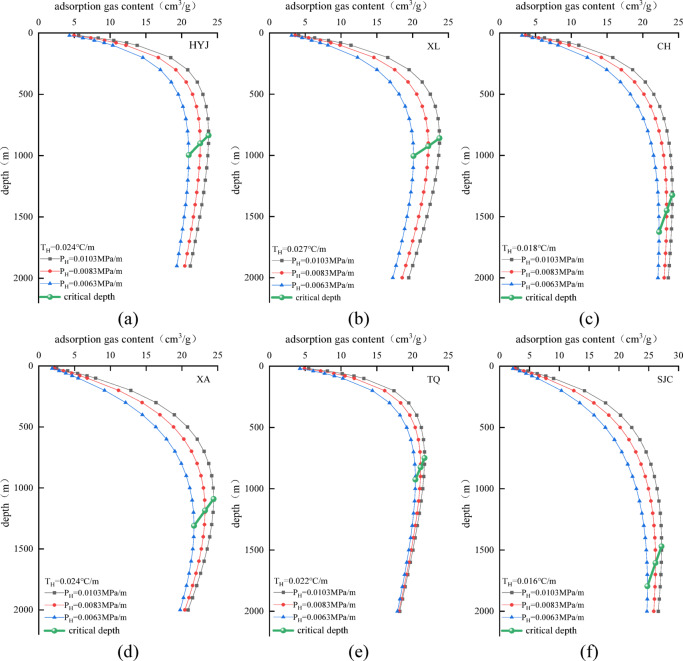
Relationship between adsorption gas content and burial depth under fluid pressure gradient constraints. (**a**) HYJ, (**b**) XL, (**c**) CH, (**d**) XA, (**e**) TQ, (**f**) SJC.

With a fixed geothermal gradient, three fluid pressure gradients were established (0.0103, 0.0083 and 0.0063 MPa/m) to plot the relationship between adsorption gas content and coalbed depth at various fluid pressure gradients (Fig. 11). At the same burial depth, the adsorption gas content of coal samples increases continuously with a rising fluid pressure gradient. Above the critical depth, the difference in adsorption gas content between different fluid pressure gradients gradually increases as burial depth increases, indicating that pressure dominates adsorption behavior. Below the critical depth, however, the adsorption gas content across different fluid pressure gradients gradually converges, reflecting the diminishing influence of the fluid pressure gradient on the adsorption gas content, with temperature becoming the dominant factor in adsorption.

**Fig. 12:**
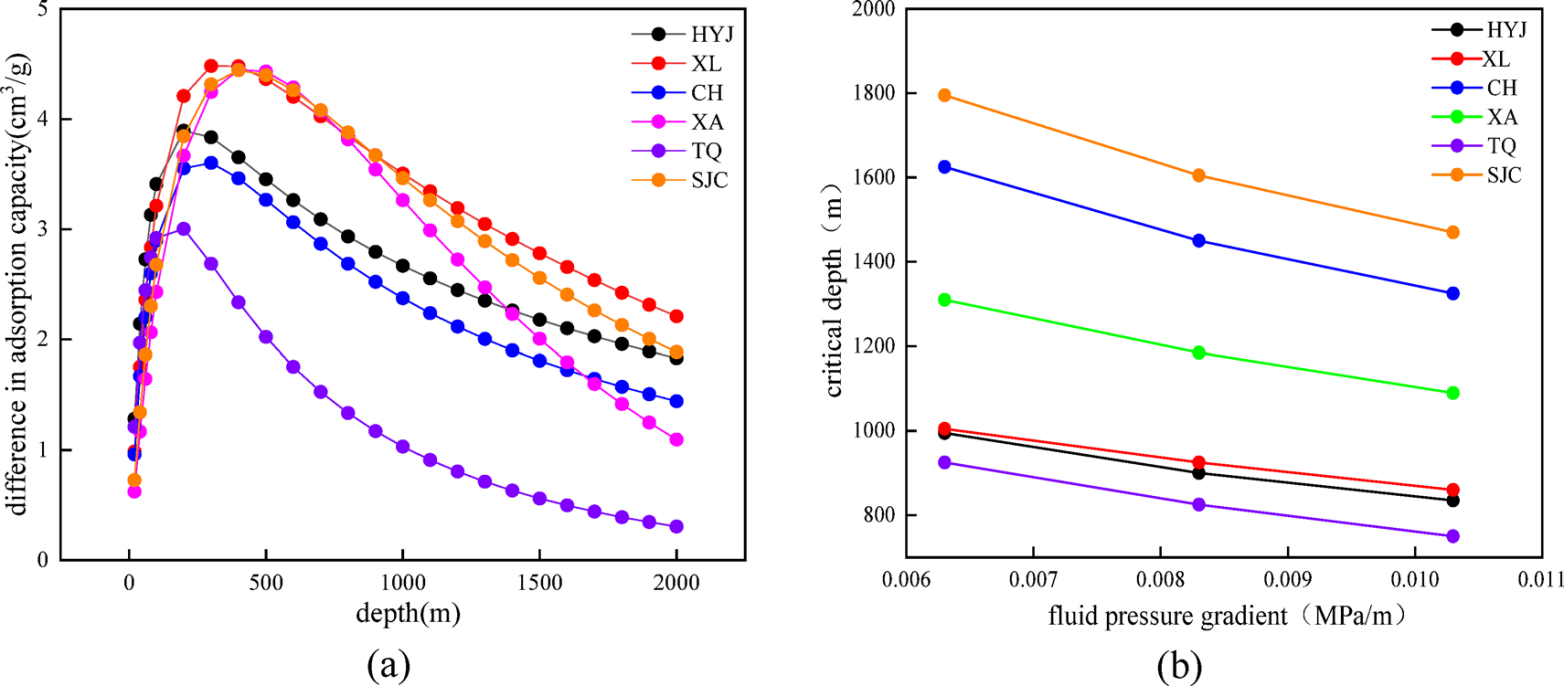
(**a**) Variation of the difference in absolute adsorption capacity with depth under different fluid pressure gradients (0.0063 MPa/m, 0.0103 MPa/m), (**b**) Relationship between fluid pressure gradient and critical depth.

For the six coal samples, the difference in absolute adsorption capacity under two fluid pressure gradients (0.0103 MPa/m and 0.0063 MPa/m) exhibited a single-peak trend of “initial increase followed by decrease” with increasing burial depth(Fig. 12a): Stage 1: Dominated by the positive pressure effect, the sensitivity of adsorption behavior to differences in pressure gradients rapidly increased with burial depth, with the difference reaching a peak in the 300–500 m range; Stage 2: As formation temperature rose, the negative temperature effect began to partially offset the contribution of pressure to adsorption, weakening the sensitivity of adsorption to changes in pressure gradients and causing the difference to continue to decline. This pattern reflects the transitional characteristics of temperature–pressure coupling above the critical depth and is consistent with the temperature–pressure dynamic equilibrium mechanism of CBM adsorption at depth.

Further investigation of the influence of the fluid pressure gradient on the critical depth (Fig. 12b): As the fluid pressure gradient increases from 0.0063 MPa/m to 0.0103 MPa/m, the critical depth of each coal sample shifts towards shallower. For instance, as the fluid pressure gradient increases, the critical depth of the HYJ coal sample decreases from 995 to 915 m.

The combined effects of fluid pressure gradient and geothermal gradient govern the critical depth for the accumulation of adsorbed gas in coalbeds, thereby defining the boundary between deep and shallow CBM resource. Pressure actively promotes the enrichment of adsorbed gas within coalbeds, while temperature facilitates the conversion of adsorbed gas into free gas. Under favorable preservation conditions, supersaturated CBM with free gas will be formed in deep coal reservoirs^36,37^.

## Conclusions

1. The S-L model consistently fits the isothermal adsorption experimental data with a coefficient of determination (R^2^) above 0.930. This indicates that the model effectively describes the adsorption process of the coal samples in the study area. The adsorption capacity of the coal samples increases with rising pressure and decreases with increasing temperature.
2. The Langmuir volume and Langmuir pressure are both functions of temperature. Based on this finding, a predictive model of absolute adsorption quantity under the combined influence of temperature and pressure was developed. Further incorporating geothermal and fluid pressure gradients, a model was established to show the relationship between adsorption gas content and burial depth. This revealed that the adsorption gas content varies non-monotonically with increasing burial depth and verified the existence of critical depth.
3. The adsorption gas content first increases and then decreases with increasing burial depth, reaching a maximum value at a critical depth. At depths shallower than the critical depths, the effect of increasing formation pressure on adsorption is dominant; at greater depths, the effect of rising formation temperature on adsorption becomes dominant. There is a negative correlation between both the geothermal and fluid pressure gradients and the critical depth.
4. There is a positive correlation between coal rank and adsorption capacity. Since the study area is dominated by high-rank coal, macerals emerge as a key intrinsic factor that governs adsorption capacity. Vitrinite content shows a positive correlation with adsorption capacity, whereas inertinite content exhibits a negative correlation.

## Data Availability

The data that support the findings of this study are available from the corresponding author upon reasonable request.
